# Temperature-Insensitive Bend Sensor Using Entirely Centered Erbium Doping in the Fiber Core

**DOI:** 10.3390/s130709536

**Published:** 2013-07-23

**Authors:** Harith Ahmad, Mohd Zamani Zulkifli, Farah Diana Muhammad, Julian Md Samangun, Hairul Azhar Abdul-Rashid, Sulaiman Wadi Harun

**Affiliations:** 1 Photonics Research Centre, University of Malaya, 50603 Kuala Lumpur, Malaysia; E-Mails: mohdzamani82@yahoo.com (M.Z.Z.); faradibah90@yahoo.com (F.D.M.); juliansamangun@gmail.com (J.M.S.); swharun@um.edu.my (S.W.H.); 2 Faculty of Engineering, Multimedia University, 63100 Cyberjaya, Selangor, Malaysia; E-Mail: hairul@mmu.edu.my

**Keywords:** bending sensor, special fiber, temperature-insensitivity, erbium-doped fiber

## Abstract

A fiber based bend sensor using a uniquely designed Bend-Sensitive Erbium Doped Fiber (BSEDF) is proposed and demonstrated. The BSEDF has two core regions, namely an undoped outer region with a diameter of about 9.38 μm encompassing a doped, inner core region with a diameter of 4.00 μm. The doped core region has about 400 ppm of an Er_2_O_3_ dopant. Pumping the BSEDF with a conventional 980 nm laser diode gives an Amplified Spontaneous Emission (ASE) spectrum spanning from 1,510 nm to over 1,560 nm at the output power level of about −58 dBm. The ASE spectrum has a peak power of −52 dBm at a central wavelength of 1,533 nm when not spooled. Spooling the BSEDF with diameters of 10 cm to 2 cm yields decreasing peak powers from −57.0 dBm to −61.8 dBm, while the central wavelength remains unchanged. The output is highly stable over time, with a low temperature sensitivity of around ∼0.005 dBm/°C, thus allowing for the development of a highly stable sensor system based in the change of the peak power alone.

## 1. Introduction

Fiber optic sensors have various industrial applications such as in monitoring of structural health [1–3], due to its advantages over electronic sensors such as being non-conducting, high sensitivity, resistivity to electromagnetic disturbance and robustness in erosive, conductive or explosive environments [4]. While most traditional applications for these sensors are focused on parameters such as temperature and pressure, other applications for these sensors also abound. Among these applications are bending sensors, which have been demonstrated in various types of fibers such as multimode, single-mode and curvature fibers [5–8]. These sensors have attracted significant attention due to its ability to measure many physical parameters such as pressures, forces, frequency, vibration and many other acoustic parameters, which can find many useful applications in sensing systems such as micro-displacement and acceleration to name but a few [9]. There have been several reports on fiber bending sensing techniques using fiber Bragg gratings (FBGs) [10,11] and long period fiber gratings (LPFGs) [12,13]. Nevertheless, the disadvantage of using fiber gratings as bending sensors is that they are also sensitive to temperature fluctuations and thus have the high additional cost of wavelength demodulation processes [1]. Several studies have therefore been made to investigate how to minimize the effect of the fiber sensitivity to the simultaneous temperature changes [14]. In addition, besides the drawback of simultaneous temperature sensitivity, most fiber bend sensor designs also use a separated gain medium and fiber sensing head, which will eventually increase the complexity of the sensor design. Thus, an alternative fiber bending sensor method with a simpler setup is becoming very necessary and would provide some advantages in terms of reducing the production and manufacturing costs.

Of late, there has been work done on the use of depressed-cladding erbium doped fibers (DC-EDF) [15,16] as a bend sensor. However, the drawback of this approach is that the peak wavelength of the amplified spontaneous emission (ASE) spectrum shifts as the bending radius decreases. It is an advantage and of great interest to have a fixed peak wavelength of the ASE spectrum with respect to the change of the bending radius as this will allow a simple detection system to be built, where a filter can be inserted to coincide with the peak wavelength, which then allows for the measurement of the output power against the bending radius.

This work describes a new type of bending sensor based on a bend sensitive erbium doped fiber (BSEDF), whereby the erbium dopants are concentrated within the inner ring of the core region, while leaving the outer region of the core undoped, but still having a higher refractive index as compared to the cladding. The erbium ions act as the source for the ASE spectrum and also as the medium for the sensing head. This design is different from the S-band DC-EDF, with a W-type refractive index configuration, having a three-layer structure—the core, depressed cladding and outer cladding—with typical dimensions of 3.5, 14.0 and 120.0 μm and refractive indices of 1.472, 1.452 and 1.457 [17]. The special fiber in this proposed work is not only temperature insensitive, but also easy to splice to single-mode fiber (SMF). Due to the location of the erbium dopant which is at the centre of the fiber core, a small bend on the fiber will induce a high transmission loss, thus making this bend sensor very sensitive towards bending as well as being temperature insensitive, with a slope efficiency of only ∼0.005 dBm/°C. To the best of authors' knowledge, this is the first design of a fiber bend sensor where both the gain medium and the sensing head are created from a similar fiber, which at the time possesses temperature insensitivity. This will be described in detail in the next section.

## 2. Experimental Section

**Figure 1.**
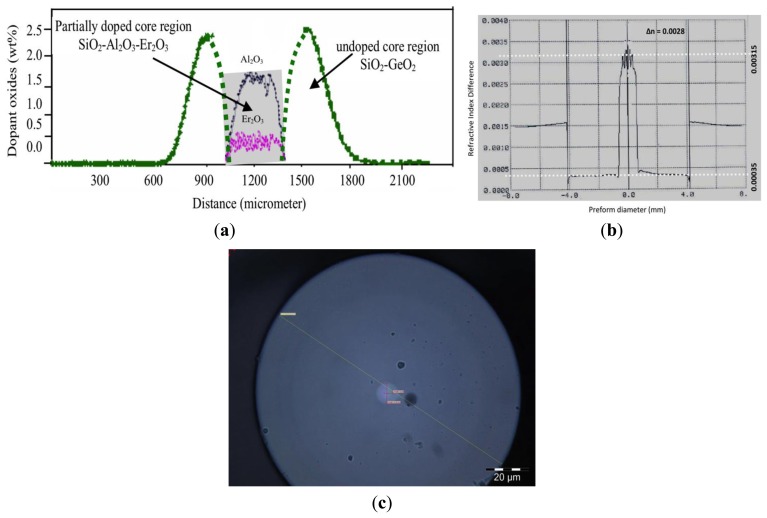
**(a)** Elemental distribution of different dopants into doped and un-doped regions of the BSEDF obtained using EPMA, **(b)** refractive index profile of fabricated BSEDF preform measured by a commercially available Preform Analyser, with a core of roughly between −0.8 to 0.8 mm and the cladding between −4.0 to 4.0 mm, with the rest being oil, and **(c)** microscopic cross section of the BSEDF.

Figure 1a shows the doping profile of the BSEDF with the center of the core doped with erbium ions as obtained from an electron probe microanalyzer (EPMA). The core of the fiber comprises of two annular regions. The outer region consists of SiO_2_, and co-doped with GeO_2_, with an external diameter of about 9.38 μm and an inner diameter of about 4.00 μm, with a refractive index of about 1.4665 and externally surrounded by a cladding layer with a dimension of 123.21 μm and a refractive index of about 1.4624, with Δ∼0.28%, which is shown in Figure 1b for the case of the fabricated preform.

**Figure 2.**
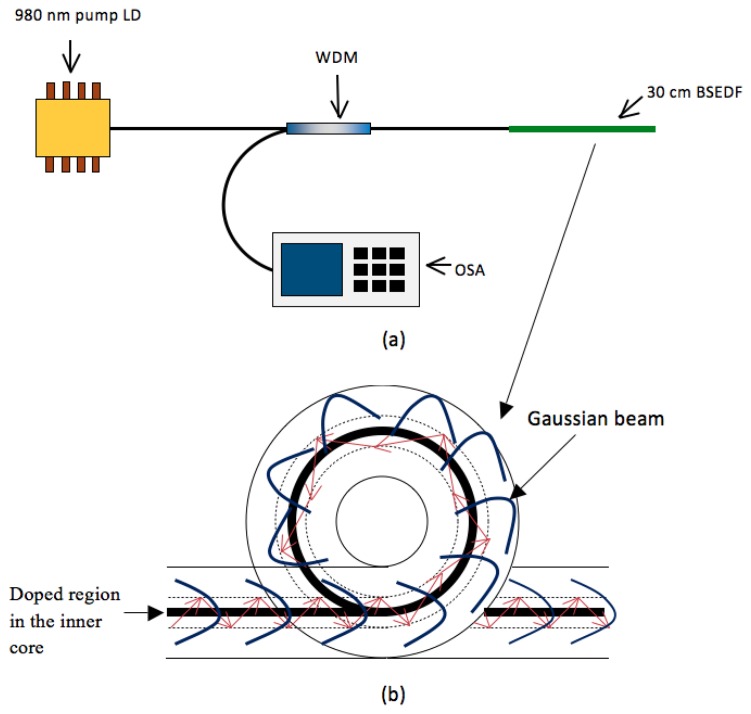
**(a)** The experimental setup of the proposed bend sensor, **(b)** The schematic diagram of the spooled BSEDF with the Gaussian beam from the LD pump and the guided rays.

The core dopant concentrations are obtained using a preform analyzer, and it is determined that the central core portion is being doped with Er_2_O_3_ of around 0.425 wt%, which is equivalent to 0.20 mole% and co-doped with Al_2_O_3_. In terms of Er ion, this is equal to 400 ppm, as shown in Figure 1a (not to scale). The central diameter region is about 4.00 μm, giving a ratio of about 0.43 between the erbium doped diameter against the core diameter. Figure 1c shows the microscopic cross section of the BSEDF. The advantage of this inner core dopant of erbium ions within the core region enhances the sensitivity of the fiber towards bending as demonstrated in this work. Figure 2a shows the experimental setup of the proposed bend sensor, which uses the BSEDF as the gain medium.

The BSEDF as described in the section above is pumped by a 980 nm laser diode operating at 50 mW and is connected to the 980 nm port of a 980/1,550 nm wavelength division multiplexer (WDM). The common-port of the WDM is then spliced to the BSEDF, which of a length of about 30 cm. The 1,550 port of the WDM is connected to a Yokogawa AQ6317 optical spectrum analyser (OSA) with a capability of giving a resolution of 0.02 nm, for spectral analysis. The BSEDF is then used as the bending sensor. As the beam from the laser diode propagates through the EDF, erbium ions are excited, hence producing the ASE that propagates in both forward and backward directions. The OSA would then detect the backward ASE spectrum that travels back through the WDM. The rationale for taking the backward ASE, instead of the forward ASE is so that there will be negligible contribution from the pump laser in the signal detection. The bend sensor mechanism is realized by spooling the EDF at different diameters from 10 cm to 2 cm and the output ASE spectrum as well as the average output power is measured against the different spooling diameters.

Figure 2b shows the schematic diagram of the spooled BSEDF with the Gaussian beam from the laser diode travelling around it. As the LD pump is injected through the fiber, Erbium ions are excited, hence producing the ASE. When the EDF is in a straight position, the propagating Gaussian beam is at its maximum intensity at the core, thus giving the excitation of erbium ions within the inner core diameter of 4.00 μm. This will give the maximum ASE emission. In the case when the EDF is bent, the ASE output with a peak wavelength of 1,533 nm will experience attenuation at even small bending radii at longer wavelengths, and augers well with the findings of [18,19]. This results in a drop in the ASE output power level. With this unique profile of the BSEDF, the power loss during the bending of the fiber can be monitored easily and changes very fast with bending. This will provide a very sensitive bend sensor. As reported in [20,21], a linear relationship between the central displacement of the beam travelling within the fiber during bending and the decrease in transmitted light intensity could be easily measured, and this is one of the parameters discussed in this paper.

## 3. Results and Discussion

**Figure 3.**
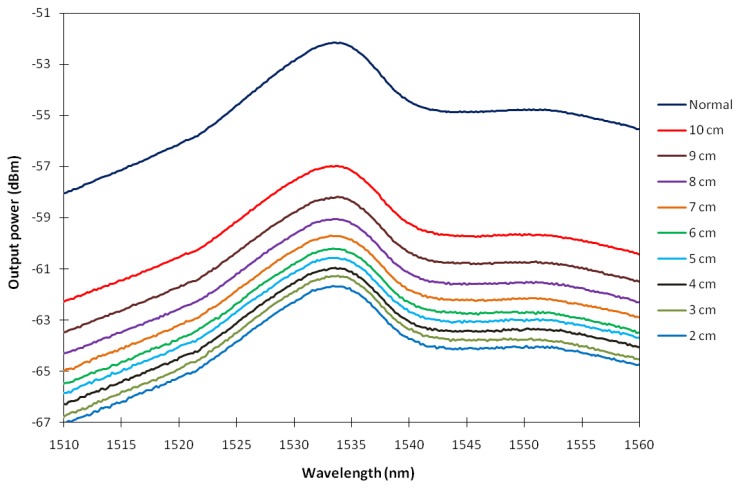
The ASE spectrum level of the BSEDF for different spooling diameter.

**Figure 4.**
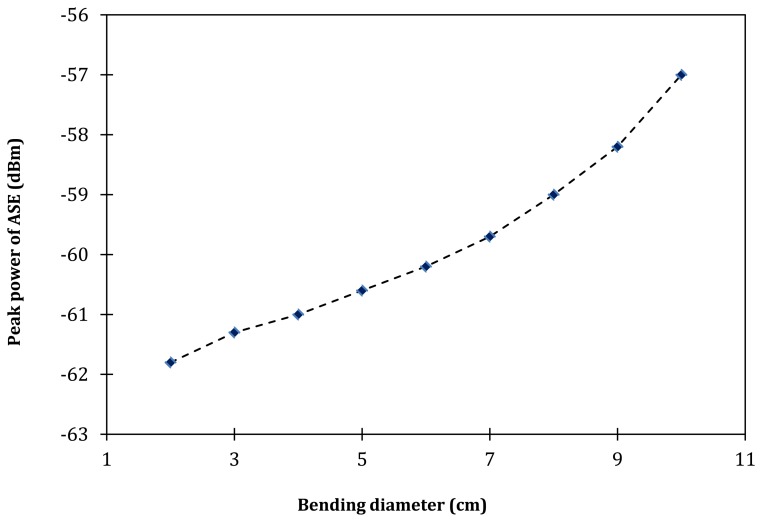
The peak power of the output ASE with respect to the bending diameter.

Figure 3 shows the ASE spectrum level with respect to different spooling diameters as taken from the OSA. As can be seen from the figure, the peak power level of the ASE spectrum also reduces with the decrease of the EDF spooling diameter, from −57.0 dBm to −61.8 dBm at the spooling diameter of 10 cm and 2 cm respectively. As the spooling diameter is reduced from 10 cm to 6 cm, the peak power decreases from −57.0 to −60.2 dBm (a total loss of 3.2 dB), whereas as the spooling diameter is further reduced from 6 to 2 cm, the peak power decreases from −60.2 to −61.8 dBm (a total loss of 1.6 dB), and this occurs at about the same peak wavelength of 1,533 nm. Thus, it can be deduced that at larger spooling diameters, which range from 6 cm to 10 cm, the loss of the peak power with respect to the decrease of the spooling diameter is higher compared to the peak power loss at smaller spooling diameters, which is within the range of 2 cm to 6 cm. This is shown in Figure 4, which shows a nearly linear slope. For the case of Depressed-Cladding Erbium Doped Fibers (DC-EDF), the peak wavelength of the ASE spectrum shifts as the bending radius decreases, as demonstrated by Rosolem *et al.* [16]. This will allow a simple detection system to be built, where a filter can be inserted to coincide the peak wavelength, which then allows for the measurement of the output power against the bending radius.

**Figure 5.**
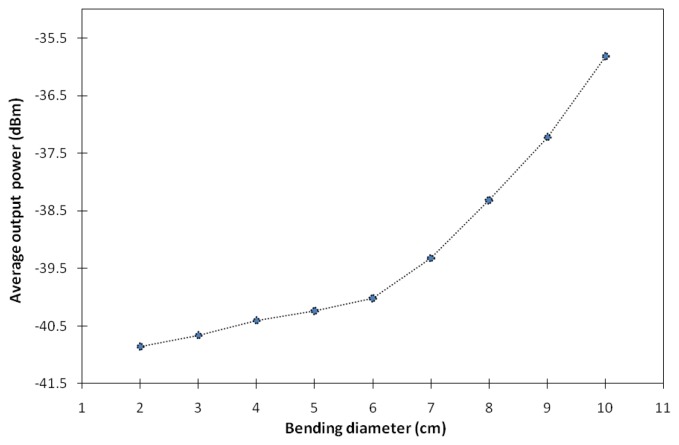
The average output power against the bending diameter.

The relationship between the bending diameter and the average output power measured from the power meter is shown in Figure 5. As can be seen from the figure, the average output power increases exponentially with the increase of the bending diameter. As the bending diameter is increased from 2 to 6 cm, the average output power increases gradually from −40.9 to −40.0 dBm, giving a slope of 0.23 dBm/cm.

On the other hand, at larger bending diameters above 6 to 10 cm, the average output power increases abruptly from −40.0 to −35.8 dBm as the bending diameter is increased, with a slope of 1.1 dBm/cm. From this point, the fiber shows higher sensitivity to bending effect as the slope gets steeper, compared with the slope for bending diameters smaller than 6 cm. Based on this characteristic, this working mechanism can be developed into a reliable and simple bending sensor requiring only a simple filter at the peak wavelength, or measuring the total output power using a simple energy detector, for a very sensitive method for measuring bends or curvature. The present measurement of the diameter is not restricted to these values, a smaller diameter can be measured, and the ASE output will still be detectable as compared to DC-EDFs, where at very small diameters the ASE output becomes negligible.

**Figure 6.**
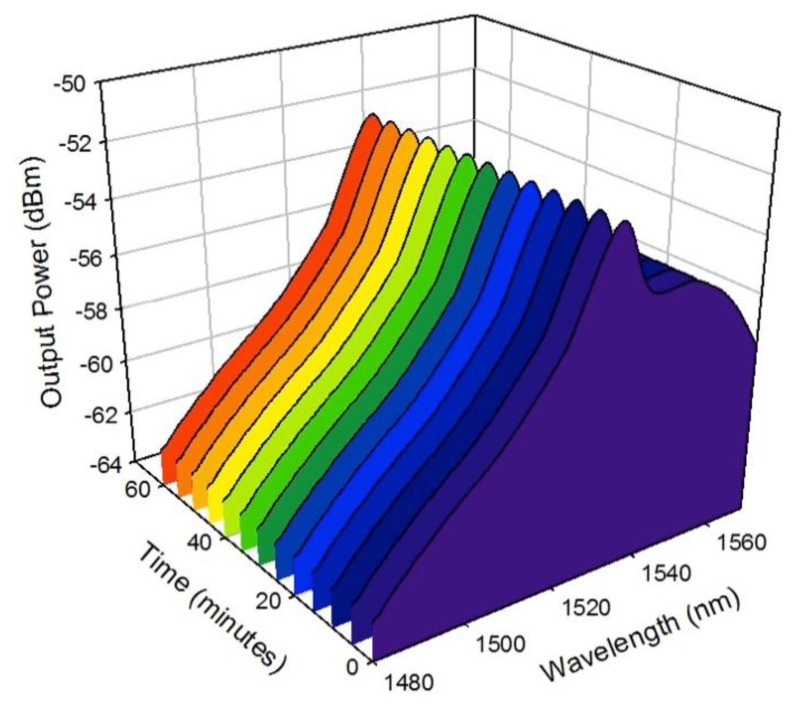
The stability measurement of the ASE spectrum within 60 min of observation time.

In order to test the stability performance of the proposed system, a stability measurement of the ASE spectrum from the BSEDF is carried out within 60 min of observation time with an interval of 5 min between the measured outputs. This measurement is taken with the BSEDF in its unspooled position and the result is shown in Figure 6. With an interval of 5 min, the output spectrum of the ASE is observed to be constant for the 60 min observation time. No significant variation is observed in the ASE spectrum in terms of the output power and the output wavelength, and similar results are obtained for spooled cases. This proves and confirms the high stability of this proposed system.

**Figure 7.**
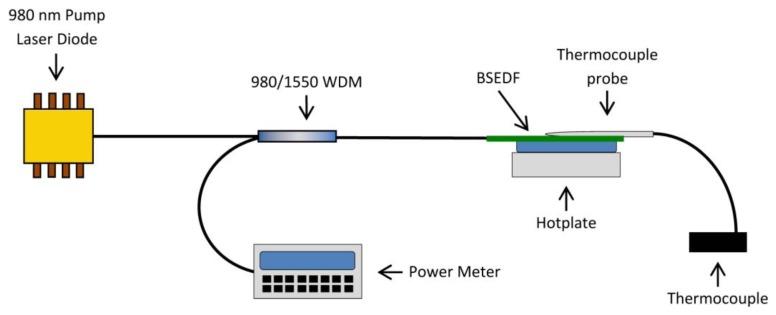
The setup for the sensitivity measurement of the BSEDF towards the temperature.

**Figure 8.**
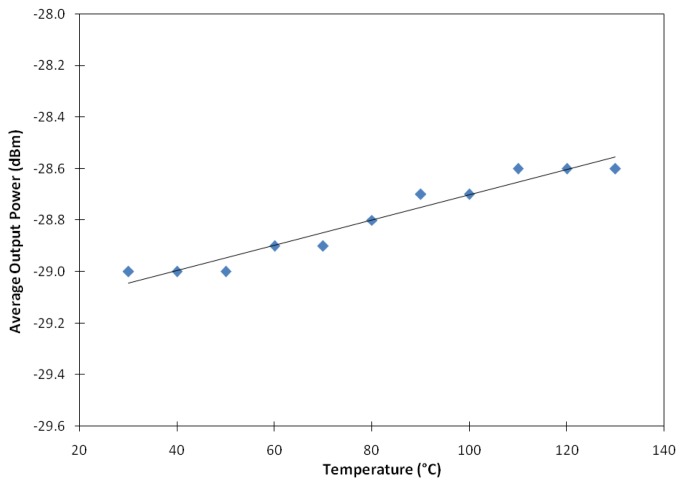
The average output power of the ASE against temperature.

Another important parameter measurement of interest is the effect of temperature on the BSEDF or the sensitivity of the BSEDF towards the temperature change. Another set-up to test the sensitivity of the fiber to different temperatures is shown in Figure 7 and the result of the test is shown in Figure 8. As seen in Figure 7, the BSEDF is laid straight on a hot plate with a variable temperature. An aluminum tape is used to make the BSEDF stick to the surface of the hot plate. Aluminum is a good thermal conductor and helps keep the temperature constant along the fiber. A power meter is used to measure the average output power of the ASE from the BSEDF.

Figure 8 shows how the average output power of the ASE from the BSEDF varies with temperature. The average output power shift against the increase of the temperature is so small that it is hardly discriminated within the temperature range from 30 to 130 °C. The relationship between the average output power and temperature indicates that this sensing system is temperature insensitive, with a slope efficiency of only ∼0.005 dBm/°C. Thus, this fiber can be said to have shown very minimum sensitivity towards temperature. Although there is a slight increment in the power meter's readings as the temperature increases, the value is too low to infer the fiber as temperature sensitive. This slight change in the reading is probably due to the air around the fiber's loose end being heated up resulting in a decrease in its reflective index. The hot air around the fiber's loose end reflects more ASE, hence giving a slight change in the power meter reading.

**Figure 9.**
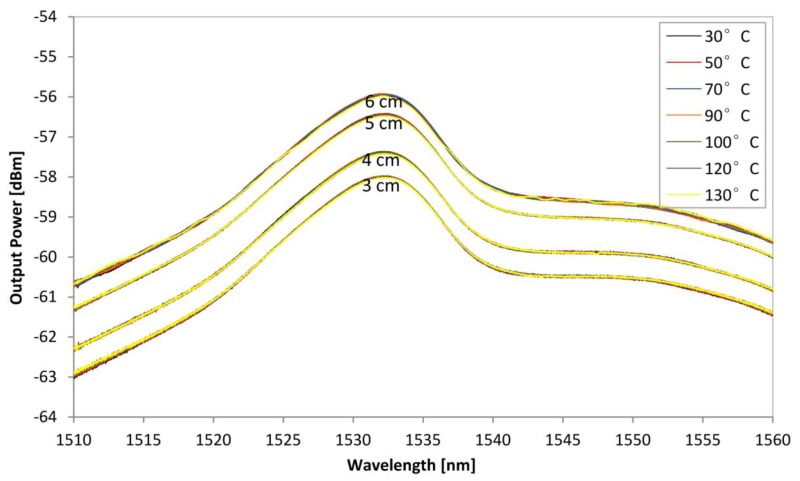
The ASE spectrum level of the BSEDF at different temperature for different spooling diameter.

The same experiment as in Figure 7 is repeated using the BSEDF in a bent condition, with different spooling diameters, replacing the power meter with the OSA as to observe the ASE output. Figure 9 shows the ASE spectrum level of the BSEDF at different temperature for spooling diameters of 3, 4, 5 and 6 cm. It is observed that there is no significant variation of the ASE spectrum level detected by changing the temperature from 30 °C to 130 °C at each different spooling diameter. For instance, in the case of 3 cm spool diameter the ASE curves at different temperatures superimpose as a single line, which indicates clearly that the bend sensor is insensitive to temperature changes.

**Figure 10.**
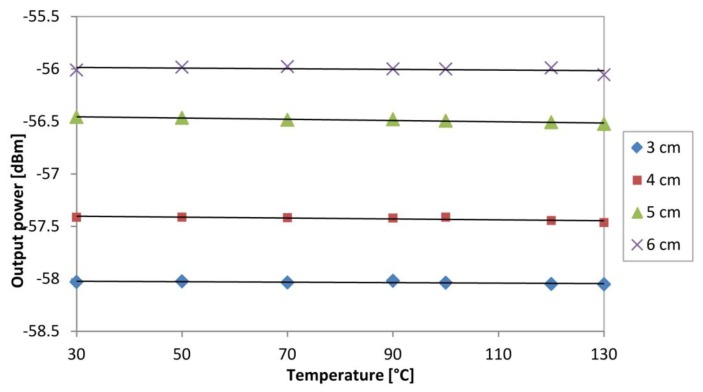
The output power at the peak wavelength of the ASE of 1,533 nm against temperature for different spooling diameter.

Besides this, it would also be of interest also to observe the output power at the peak wavelength, 1,533 nm of the ASE spectrum when exposed to different temperatures. This is done for different spooling diameters, and this is shown in Figure 10. As before, no observable changes can be seen in the 1,533 nm power at different temperatures, thereby validating the fact that the sensor is insensitive to temperature, especially at the 1,533 nm region.

In comparison to other types of bend sensors, for instance in the DC-EDF, bend-sensor with FBG and normal single-mode fiber, there tend to be a sizeable dependence on the temperature, thereby giving results that need to be compensated. The ability of the proposed sensor to cancel out cross sensitivity to other parameters is characteristic of a good sensor.

## 4. Conclusions/Outlook

In this work, a bend sensor using a uniquely developed BSEDF is proposed and demonstrated. The BSEDF has a core with an un-doped outer region of about 9.38 μm and inner core region of about 4.00 μm with an Er_2_O_3_ dopant concentration of 400 ppm. The refractive index difference between the core and cladding is about 0.28%. The BSEDF is pumped by a 980 nm laser diode at 50 mW, and when unspooled emits an ASE spectrum spanning from 1,510 to over 1,560 nm at the output power level of −58 dBm, with a central wavelength of 1,533 nm and a peak power of −52 dBm. Spooling the BSEDF decreases the peak power, from an average of −57.0 dBm to −61.8 dBm at spooling diameters of 10 cm to 2 cm. However, the central wavelength remains unchanged, thus allowing for a simple sensor to be built based on the variation of the peak power alone. The output of the ASE is also highly stable, with no observable variation in the power output over a measurement period of one hour. The BSEDF is also temperature insensitive, with only a minor variation of about ∼0.005 dBm/°C measured, thus reducing the effects of cross-sensitivity.
